# Drivers of MERS-CoV Emergence in Qatar

**DOI:** 10.3390/v11010022

**Published:** 2018-12-31

**Authors:** Elmoubasher Farag, Reina S. Sikkema, Tinka Vinks, Md Mazharul Islam, Mohamed Nour, Hamad Al-Romaihi, Mohammed Al Thani, Muzzamil Atta, Farhoud H. Alhajri, Salih Al-Marri, Mohd AlHajri, Chantal Reusken, Marion Koopmans

**Affiliations:** 1 Ministry of Public of Health, Doha 42, Qatar; mnour@moph.gov.qa (M.N.); halromaihi@moph.gov.qa (H.A.-R.); malthani@moph.gov.qa (M.A.T.); dralmarri@moph.gov.qa (S.A.-M.); malhajri1@moph.gov.qa (M.A.); 2 Department of Viroscience, Erasmus University Medical Center, Wytemaweg 80, 3015 CN Rotterdam, The Netherlands; c.reusken@erasmusmc.nl (C.R.); m.koopmans@erasmusmc.nl (M.K.); 3 Division Veterinary Public Health, Institute of Risk Assessment Sciences, Faculty of Veterinary Medicine, Yalelaan 2, 3584 CM Utrecht, The Netherlands; tinkavinks@gmail.com; 4 Department of Animal Resources, Ministry of Municipality and Environment, Doha 35081, Qatar; walidbdvet@gmail.com (M.M.I.); muzamilata@yahoo.com (M.A.); m6066@mme.gov.qa (F.H.A.)

**Keywords:** Drivers, MERS-CoV, Qatar

## Abstract

MERS-CoV (Middle East respiratory syndrome corona virus) antibodies were detected in camels since 1983, but the first human case was only detected in 2012. This study sought to identify and quantify possible drivers for the MERS-CoV emergence and spillover to humans. A list of potential human, animal and environmental drivers for disease emergence were identified from literature. Trends in possible drivers were analyzed from national and international databases, and through structured interviews with experts in Qatar. The discovery and exploitation of oil and gas led to a 5-fold increase in Qatar GDP coupled with a 7-fold population growth in the past 30 years. The lifestyle gradually transformed from Bedouin life to urban sedentary life, along with a sharp increase in obesity and other comorbidities. Owing to substantial governmental support, camel husbandry and competitions flourished, exacerbating the already rapidly occurring desertification that forced banning of free grazing in 2005. Consequently, camels were housed in compact barns alongside their workers. The transition in husbandry leading to high density camel farming along with increased exposure to humans, combined with the increase of camel movement for the racing and breeding industry, have led to a convergence of factors driving spillover of MERS-CoV from camels to humans.

## 1. Introduction

Emerging infectious diseases are a cause for increasing global concern, because of their impact on global health and economics [1]. The Ebola outbreak in West Africa during 2014-2015 showed that pathogens which previously caused small and easy to control outbreaks had the potential to infect thousands of people under the right circumstances [2]. This is also a concern for the Middle East Respiratory Syndrome coronavirus (MERS-CoV), which until now has been the cause of sporadic cases and hospital outbreaks [3]. To date, there have been 2220 confirmed laboratory cases worldwide, with 790 deaths [4]. All MERS index cases are linked to the Arabian Peninsula. Dromedary camels have been identified as a reservoir of MERS-CoV with occasional zoonotic transmission to humans [5,6]. Human-to-human transmission is also common, with around 30% of the MERS cases reported to WHO being health care associated [7,8]. However, the source of infection of many index cases remains unclear [9,10].

Studies have shown that MERS-CoV, or related viruses have been circulating among camels at least since 1983 [11]. Since that period, massive changes have occurred in people’s lives and in animal husbandry across the Arabian Peninsula. Understanding these changes may help to reconstruct the events that led to the emergence of MERS-CoV as a human disease. Past research identified several drivers of emerging zoonoses, such as urbanisation, population growth and demography, and environmental and agricultural changes [12,13,14]. The drivers which could have potentially influenced the MERS-CoV emergence in humans have only sporadically been investigated [15,16]. By reviewing changes involving humans and camels over the past 30 years in Qatar, this study sought to identify the key drivers of the emergence and spread of MERS-CoV.

## 2. Methods

Potential drivers for disease emergence were identified from literature and from discussions with national and international experts in MERS-CoV. The final list had the following categories: economic development; human demography and behavior; international travel, commerce, sports and leisure; political environment; agriculture and food industry change, including camel demography, husbandry and movement; changes in climate and land use. Data from 1980 onwards were collected from national and international databases. If multiple data sources were available, data from both sources were collected. All data were entered in an excel datasheet and reviewed and discussed with the project team (Supplementary 1).

Qualitative information and remaining data gaps were addressed by interviews with a group of 15 experts and stakeholders from Qatar. Criteria to select experts included 5 years or more experience in a camel-related business (farming, trading and racing) or professional services related to camels and being familiar with cultural aspects of the Qatari community. Using a structured interview guide (Supplementary 2) and a moderator, a series of 4 interviews were conducted in Arabic, each lasting approximately for 3 hours. The main themes that were covered during the interviews included: (changes in) people’s living conditions; customs and purposes of camel ownership; cultural habits related to camels; educational level and personal behaviors of camel owners and workers; camel movement; demographic distribution of camels in Qatar; camel farming practices: feeding, grazing, and slaughter. A detailed transcript was shared with the experts for authentication. A literature search was done to complement findings from the quantitative and qualitative study, using PubMed, Google Scholar and the local sources of information including the Ministry of Public Health (MoPH), Ministry of Municipality and Environment (MME), Ministry of Development and Planning Statistics (MDPS), and Qatar Statistical Authority (QSA).

The funder had no role in study design, data analysis, data interpretation, or writing of the review.

## 3. Results

### 3.1. Changes in the Economic Situation

**Figure 1:**
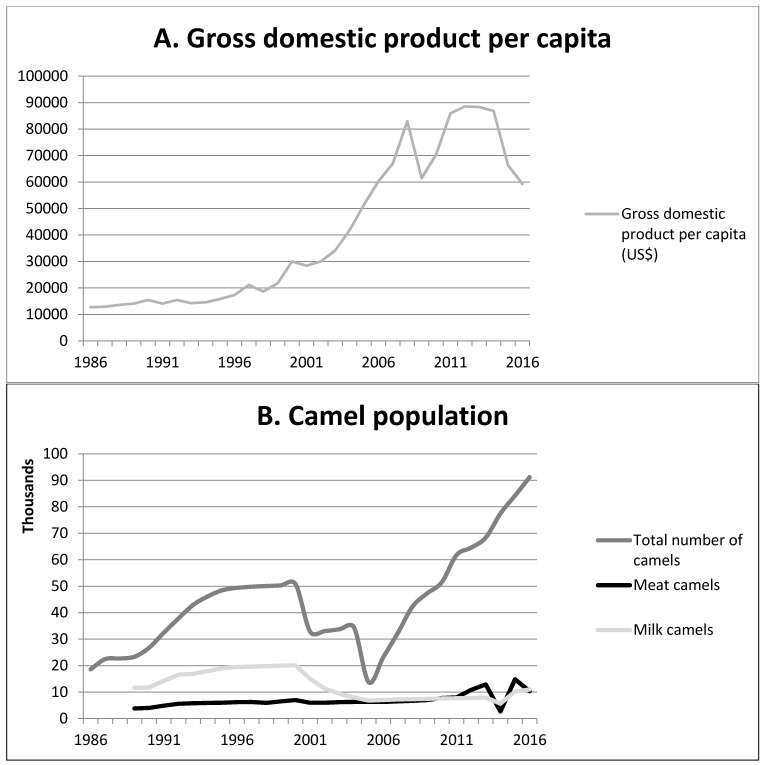

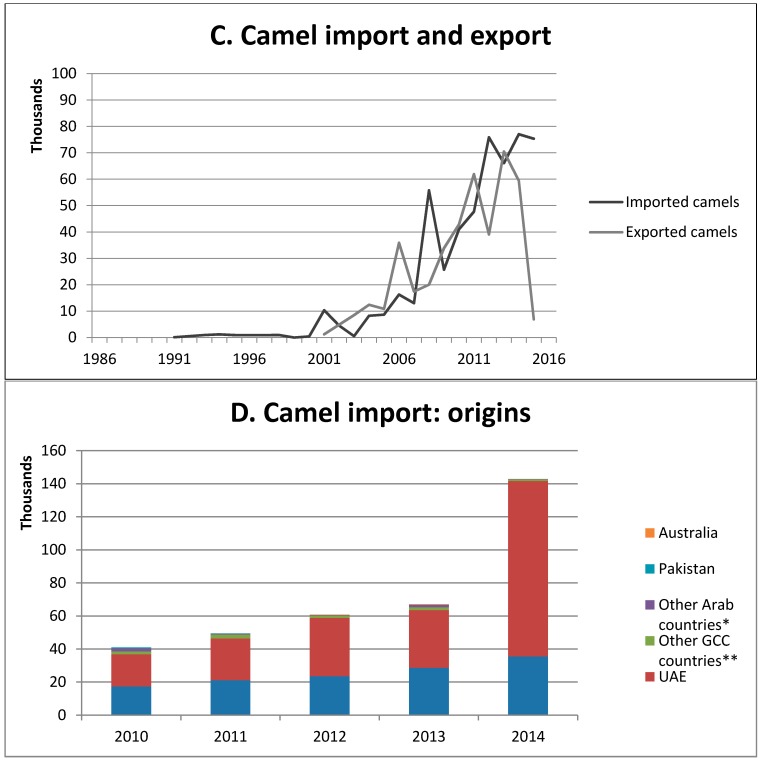
Developments in economy, camel demography, production, and trade. (**A)**. Development over time of the gross domestic product per capita; (**B**) Development over time of the camel population; (**C**) Development over time of camel import and export; (**D**) Development over time of camel importation per country of origin. *Other Arab countries: Algeria, Comoros, Djibouti, Egypt, Iraq, Jordan, Lebanon, Libya, Mauritania, Morocco, Palestine, Somalia, Sudan, Syria, Tunisia, and Yemen. **Other GCC countries: Bahrain, Kuwait, Oman, Qatar.

Historically, Qatari inhabitants were mostly Bedouins along with a few settled people [17,18]. The Bedouins owned limited numbers of camels, sheep, and goats [19]. Camels were used as a source of food (milk and meat) and means for transportation. In 1939, oil and natural gas resources were discovered. However, large-scale exploitation started in the 1950s [20]. From the 1950s onwards, Qatar’s economy has been steadily growing. However, the year 2000 marked a significant turning point as Qatar’s GDP almost increased by more than 5-fold during the period 2000–2006 (Figure 1A) [20,21]. Qatar is currently considered to be one of the wealthiest countries in the world [20].

### 3.2. Changes in Human Demography and Health

**Figure 2:**
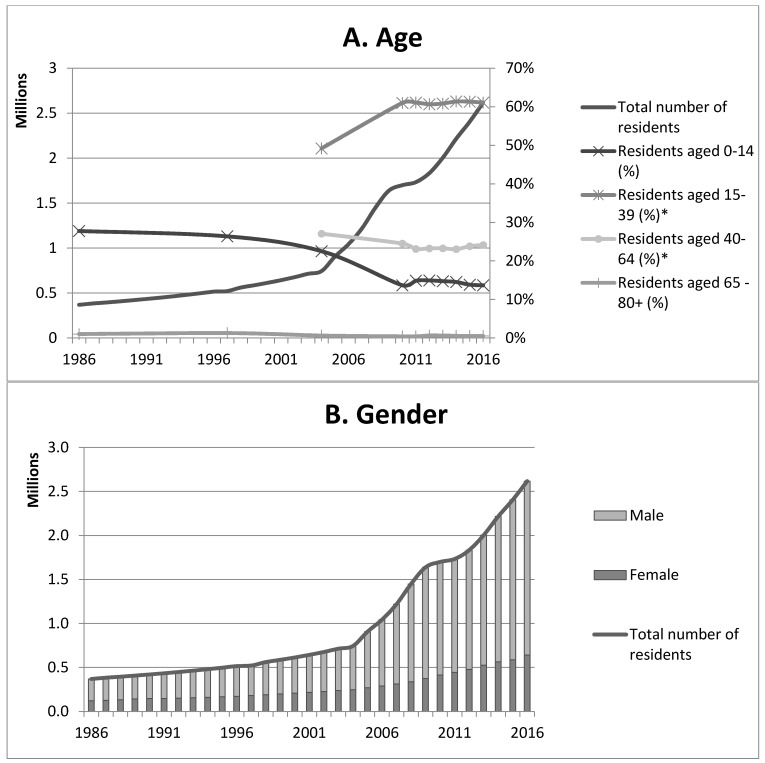

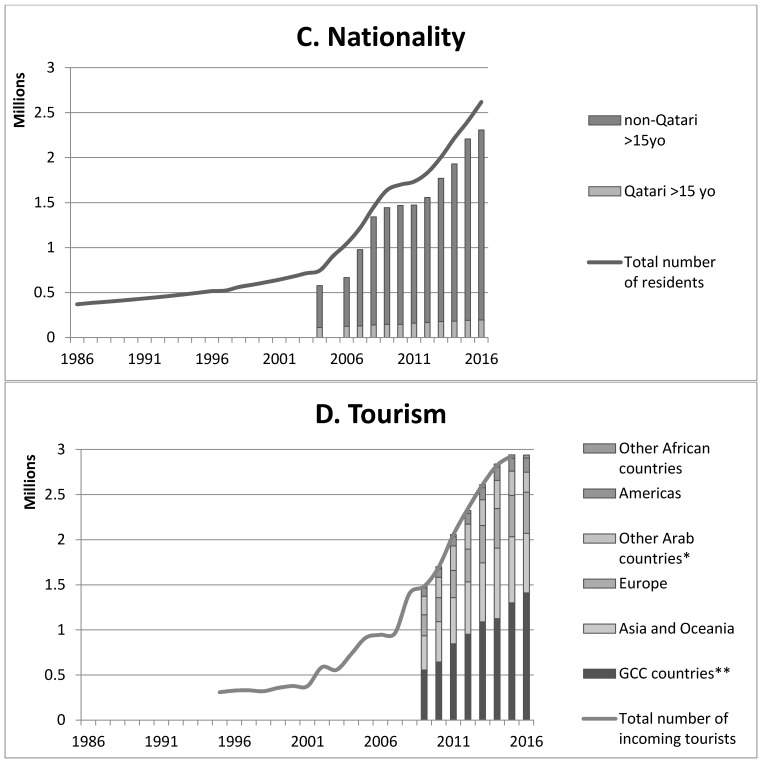
Developments in human demography in Qatar; (**A**) Development over time of the age structure of the population; (**B**) Development over time of the human sex ratio; (**C**) Development over time of the ration Qatari vs. non Qatari; (**D**) Development over time of tourism per country of origin *Other Arab countries: Algeria, Comoros, Djibouti, Egypt, Iraq, Jordan, Lebanon, Libya, Mauritania, Morocco, Palestine, Somalia, Sudan, Syria, Tunisia, and Yemen. **GCC countries: Bahrain, Kuwait, Oman, Qatar, KSA, and the United Arab Emirates.

The thriving economy was paralleled by major demographic and life style changes. In the late 1950s, around 16,000 people lived in Qatar [22]. In response to demands for a larger workforce after the exploitation of oil and gas began, foreign laborers started to migrate to Qatar from countries in the region, like Palestine, Oman, Iran, and the Kingdom of Saudi Arabia (KSA). Later, immigrants from Pakistan, India, Nepal, Sri Lanka, Bangladesh, the Philippines, and Indonesia joined the older migrant populations, increasing the number of inhabitants to 369,079 by 1986 and recently to 2,617,634 (Figure 2A) [23]. In 2016, non-Qatari males made up 78% of the residents of working age (15-64) and non-Qatari made up more than 90% of the total number of Qatar inhabitants older than 15 years of age (Figure 2B,C) [24]. Most recent estimations of the origins of the non-Qatari population are that 25% is Indian, 11% Bangladeshi, 14% Nepali, 10% Filipinos, 9% Egyptian, 5% Pakistani, and 2% Iranian [25]. The total number of males in Qatar increased from 67.2% of the total population in 1986 to 75.5% in 2016 (Figure 2B). In 2004, almost 50% of residents were between 15 and 39 years old, and this has risen to more than 60% in 2015 (Figure 2A). Detailed accounts on age distribution were not available before 2004 [26].

Most people in Qatar live in urban areas. The percentage of residents living in cities increased from 85.3% in 1960, to 90.4% in 1986, and 99.3% in 2016 [20]. Doha, the capital and the biggest city of Qatar, hosts the greatest number of people. However, there has also been a large increase in number of people living in the Al-Rayan area, where most of the camel farms are located. The number of tourists visiting Qatar also increased, especially since 2000. Most tourists came from other GCC countries, but the number of visitors from Europe and America were also increasing (Figure 2D) [20,27].

According to experts, the economic development and population increase coincided with major changes in life style. The Bedouin nomadic lifestyle gradually decreased as most of the Qatari tribes shifted to an urban, settled lifestyle; cars and planes rapidly replaced camels as transportation means. This transformation to a more sedentary lifestyle is reflected in the profile of comorbidities. More than 70% of adults are overweight and almost half of them obese [28]. Male obesity increased from 17% in 1986 to 34% in 2014, which is extremely high compared to the current 11% prevalence in men worldwide [29]. In 1998, 7% of residents above 15 years were hypertensive, rising to 14% in 2006, and 33% in 2012 [30,31]. Prevalence of high blood sugar among adults in 2015 was 14%, compared to a worldwide prevalence of 9% [28]. The Qatar Stepwise Report reported in 2012 that 15% of adults were daily smokers. Yet, Qatar has a low death rate: 1.49/1000, compared to the worldwide death rate of 7.72/1000, and its healthcare system has developed rapidly over the past twenty years [28,31,32].

### 3.3. Changes in Camel Husbandry and Practices

The increase in the number of dromedary camels reflects the increasing popularity of camels as sports animals (Figure 1B). With the changing life style and increasing wealth, the purchase and breeding of (expensive) racing camels came within reach of an increasingly large segment of the Qatari national population. According to experts, although camel racing has traditionally been part of the Bedouin culture, the organized racing business went through major changes over the past decades. This was partly due to financial and regulatory support from the Qatari government. This support increased the social and economic value of camels in Qatar, further stimulating their popularity. The Al-Shehaniya camel-racing track, one of the biggest tracks in the Gulf, was opened in 1990 [33]. The camel farms that are located near the Al-Shehaniya camel racing area are mostly used for racing camels. There are about 1500 racing camel holdings at the Al-Shehaniya camel racing area. Some of the camels in Qatar are used to compete in camel beauty contests that are organized around the Arabian Peninsula. According to the FAO, in 1960 there were about 6,000 camels in Qatar. This rose to over 43,000 in 1992, 50,000 in 2000, and more than 90,000 in 2016 (Figure 1B). More than 83% of the animals are currently kept for racing [34]. Across the Gulf region, Qatar has the highest camel density, with 6.77 units/km^2^, compared to 4.74 units/km^2^ in United Arab Emirates (UAE) and 0.11 units/km^2^ in the KSA [35]. In 2005, the total number of camel farms was 1300 and by 2014 it had increased to 9594 [34].

**Figure 3:**
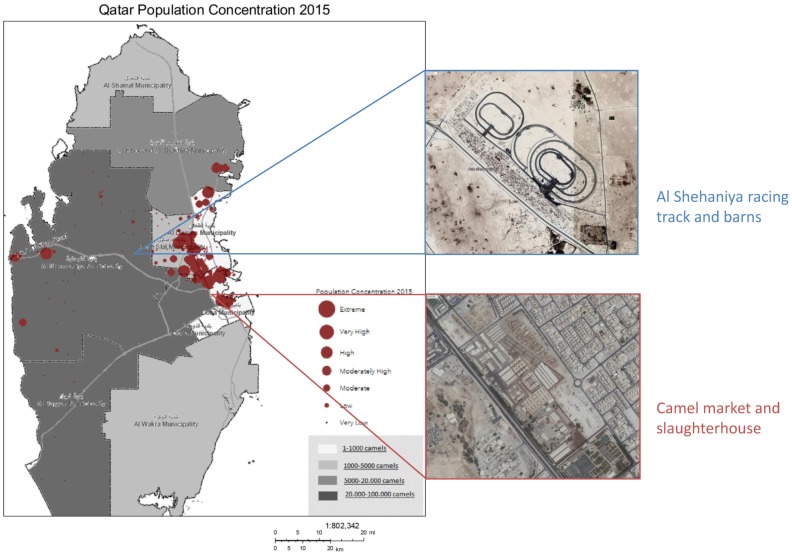
Human and camel density map of Qatar (adapted from Ministry of Development Planning and Statistics, Population Concentration map, 2015). The density map shows the density of camels (source: Ministry of Municipality and Environment) and humans in Qatar. Most people live in and around Doha, where the Doha animal market and slaughterhouse are also located. The highest camel density can be found in the Al-Rayyan area, where the Al-Shehaniya racing tracks are also located. A small, but growing, part of the Qatar population also lives in the Al-Rayyan area.

As a result of the loss of traditional methods of rangeland management, the vegetation coverage decreased from 10% to only 1% of total land cover. Overgrazing of the green areas due to the increased population of camels and other livestock accelerated the desertification of Qatar [36]. Therefore, the government decided to assign natural protected areas in 2004 [37,38], and started to sanction the free grazing of livestock since 2005 [39]. By 2011, open grazing was completely banned [40]. According to the experts’ opinions, this led to changes in farming practices, as herds were then moved outside of Qatar to areas where free grazing remained possible. Moreover, in Qatar, camels are now raised in closed systems and within 1 of 9 designated farming areas (camel complexes) in the residential districts. Camel workers also live on the premises of the camel complexes. Typically, a camel complex has a reception room (Majlis) for social activities of the camel owners. The Al-Rayyan municipality, where the Al-Shehaniya camel racing area is also located, currently holds about 83% of the total camel population and 61% of camel holdingss (Figure 3) [34,35]. According to the experts, this newly adopted closed farming system led to the increase of disease incidence, especially of parasitic diseases. However, we did not find any disease statistics to substantiate these findings.

### 3.4. Changes in Race Camel Farming and Practices

**Figure 4:**
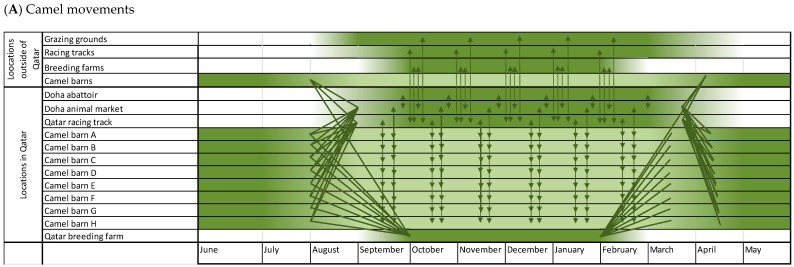

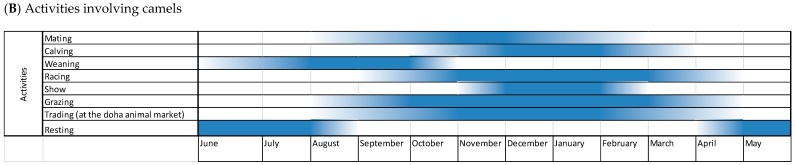
Seasonality and movements of camels and camel activities. (**A**) Dark green locations and periods of time indicate a high concentration of camels. The arrows show the direction of camel movements. It is shown that many camels gather and mix at the animal market, racing track, and breeding farms in Qatar in August, September and October. Moreover, there is constant movement to and from grazing grounds and racing tracks outside of Qatar. In March and April, most camels travel back to their barns. (**B**) shows the seasonality of camel related activities. Most activities take place in the “cold season” from September to April.

The increasing focus on camel race competitions caused big changes in camel farming practices. Previously, the calves were weaned when the next calf was born. Currently, weaning occurs at around 7 months of age. After being weaned, young camels are directly taken for acclimatization (during the period mid-July through mid-August) from the general livestock farms (located across the region) to the racing farms, mostly located within the Al-Shehaniya area. This involves drastic changes in feeding systems, intense training for races, and mock races alongside camels from other farms and older training camels. The off-season for camel racing is during summer (mid-April to August) (Figure 4). During this time, most of the owners travel abroad, the frequency of visits to the farms substantially decreases, and workers are permitted to take annual vacations. From September onward, training intensifies, in preparation of the racing season, which lasts from mid-September through mid-April. During that time, 14,000 registered camels from different origins, ages, gender, nationalities, and breeds compete together at the Al-Shehaniya camel-racing track. During the racing season, up to 24 rounds take place, approximately five days per week.

### 3.5. Changes in International Camel Movements and Travel

An unprecedented, increasingly intensified mobility of camels inside and outside Qatar has been seen over the recent decades. The domestic and cross border mobility does not only involve camels, but also people who look after the camels to provide care along the journey. Import and export of camels have especially increased since the year 2000 (Figure 1C). The imported camels mainly come from the UAE and KSA (Figure 1D).

The dynamics and travel patterns of Qatari camels are complex (Figure 4). Camels are transported to and from different locations, for a variety of purposes, and with a noticeable seasonal pattern. Mobility gets more intensive during the racing and trading season (September to April). Experts believe that the ban of open grazing in Qatar played a key role in the intensity and frequency of camel movements. They mention that there has been a remarkable increase after 2011 in numbers of camel workers and owners who cross the borders to and from KSA along with their animals, although this recently stopped with the KSA-Qatar political situation. The ban of open grazing stimulated camel owners to establish farms in KSA and UAE where open grazing is still permitted. Therefore, camels are moved through Gulf Countries, particularly during the winter season.

Camel races and beauty contests that are routinely organized in nearly all Gulf countries are another factor that boost the national and international movement of camels. Compared to other types of camels, racing camels dominate in terms of numbers and frequency of mobility both across borders and domestically, particularly between September and April. As per the records of the Camel Racing Committee, in the 2016 racing competitions, 14,000 camels from Qatar and camels from the other GCC countries contested [35]. However, owing to the lack of standardized identification system, it was difficult to determine the exact figures and the extent of these movements.

Camels are also being mobilized for reproduction purposes (Figure 4). Mating season (also known as camels’ honeymoon) starts in the middle of August and continues through February of the next year, with the high season in the September-October period. Female camels are usually taken from their own location to other farms where selected males are kept particularly for reproduction purposes. About 14,000 female camels are annually being moved for mating. They spend around 1 week at a breeding farm with male camels before they are taken back to their original farms. Programmed mating is exclusively being practiced for race and show camels. The mating season is another seasonal activity that entails intensive movements of camels, camel owners, workers, car drivers and veterinarians.

### 3.6. Changes in Camel Trade

The Doha wholesale market constitutes the primary hub for camel trading. In parallel with the increased number of camel races, Al-Shehaniya City also grew as a market and has become a hub for trade of racing and beauty show camels in Qatar. The wholesale market in Doha hosts camels and other types of livestock from countries all over the Gulf region. The camels typically stay at the market until they are sold. Camel workers live at the market premises. Camels that are being sold (calves in particular) serve a variety of purposes. They are sold to be slaughtered at the Doha wholesale market abattoir, for breeding purposes, to be trained as racing camel, or to be prepared for camel show competitions. 

In recent years, the Doha wholesale market has been surrounded by rapidly growing residential areas. Animals in the market are now in close proximity to the residents. As of 2005, slaughter practices were banned inside residential premises, and can only be performed in official slaughterhouses and exclusively by licensed persons.

### 3.7. Changes in Use of Camel Meat, Milk, and Urine

Camel meat and milk are no longer part of the daily diet of most Qatar inhabitants. Nonetheless, camel meat is a fundamental ingredient of Qatari social events and family celebrations. Production of camel meat and milk has remained stable in the past 30 years. Camel milk is generally kept for personal use, particularly for the perceived therapeutic merits of raw camel milk, as well as camel urine. Experts state that there is an unshakable belief that the regular consumption of camel milk helps to prevent and control diabetes. It is also widely believed in the Qatari community that camel urine and milk can heal skin lesions and other diseases. Camel urine is also regularly used to whiten the skin and face and lighten the hair. The majority of camel owners offer camel milk and urine for free, as a practice of generosity.

## 4. Discussion

The role of camels in the transmission of MERS-CoV is well documented [5]. Despite the fact that MERS antibodies have already been detected in camels since 1983 [11] and human contact with animals is not new, human MERS cases were only detected in 2012 [41]. Based on institutional and literature data and in-depth interviews with key professionals, this study sought to examine the changes involving the human, animal, and environmental drivers that may have contributed to the spread and virus spillover to humans.

**Figure 5:**
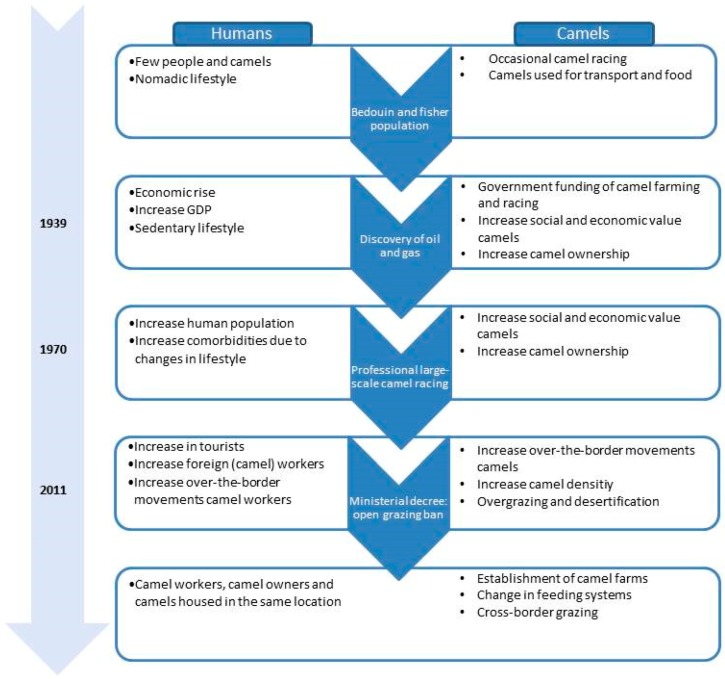
Visual summary/timeline of relevant events.

Our reconstruction of events over the past decades, based on available literature, statistics, and expert opinions, lead to the conclusion that the discovery of oil and natural gas resources has been the starting point of a chain of events that ultimately led to conditions favoring the emergence of MERS-CoV (Figure 5). This discovery led to massive economic growth. Owning a camel represents the wealth and status of its owner in Arabic culture. Governmental sponsorship of camel ownership and camel racing further stimulated the camel industry, especially the camel-racing sector. This in turn lead to an accelerating increase of the camel population, a change in camel farming, and a concomitant increase in the number of camel workers [34]. The human population of Qatar has increased by 7-fold over the last decades [23]. This is unlike other high-income countries, that have a yearly overall population growth of only 0.6% [20]. Population growth and high population density have been shown previously to be important risk factors for disease emergence [42]. Moreover, consistent with the disease profile of wealthy countries where sedentary lifestyle prevails, the prevalence of chronic diseases increased in Qatar in accordance with the increasing GPD, ultimately rendering the Qatar population not only vulnerable to virus transmission, but also to its deadly complications [43].

The intimate nature and number of interactions between camels and humans has also increased significantly in the past 30 years, increasing the risk of any zoonotic spillover. At camel complexes, workers intimately reside, sleep, and eat with their camels. Camel owners, on the other hand, pay regular visits to their barns and stay there for considerable hours every day (even longer during weekends, holidays, and winter season) in the majlis built at the corner of their barns. Owners, who are often of advanced age with multiple comorbidities, enjoy drinking fresh camel milk and entertaining guests. Those who suffer certain diseases tend to visit the camel barns to use camel urine or drink fresh camel milk for its perceived curative properties.

Among the variety of changes that involved camel husbandry in Qatar, the shift from open grazing to close housing systems seems to be most significant. Opportunities for camel-to-camel and camel-to-human spread have greatly increased since then. It is possible that housing camels in barns, with poor biosecurity and hygienic standards, turned these barns into ‘melting pots’ for the virus that ultimately acquired the ability to cross the human-animal barrier. The increase of cross-border movement of camels increased chances and frequency of (international) virus spread. Camels are transported freely across borders for a variety of purposes through multiple routes and means of transportation. When camels and the humans that accompany them, arrive at the site of a race or beauty event in Qatar, they are housed with the local camels. Owners are welcomed in the majlis at the camel complexes. The mixing of camel and human of different origins further increase chances of virus transmission.

Although much effort was made to study MERS-CoV viral sequences and MERS-CoV transmission between dromedary camels and humans, it is still unknown which genetic mechanisms have caused the viral spillover of dromedary camels to humans. However, the most important determinant of host specificity seems to be the Spike S1 protein, that recognizes and binds to host-cell receptor DPP4 [44]. Recently it has been shown that the MERS-CoV spike can rapidly adapt to species variation in DPP4 [45]. As such, the increasing human-animal interface that is described in this paper may have facilitated the adaptation of the spike protein to human DDP4. However, much remains unknown, also in view of the findings that MERS-CoV from East Africa were not phenotypically different from the viruses from the Middle East, while human MERS patients have not been reported from the African continent [46]. 

Finally, the changes in animal husbandry practices, earlier weaning, frequent grouping and transportation of animals, and the introduction of an entirely new feeding system, may induce stress in the camels. These changes and movements often involve young weaned animals, at the same time as maternal antibodies are waning, which are linked to the shedding of the virus [42,47]. Most of the limitations of this study were related to the availability of data. Firstly, statistics on animals, import and export, animal workers, and land use were only found since 2000 onwards, limiting the chance to study the trends and changes prior to that year. Secondly, even the available national data on the animals, humans, and environment were found to be sometimes inconsistent, limiting the possibility to provide “hard evidence” of causality. Nevertheless, this is the first comprehensive quantitative overview of possible drivers of MERS-CoV in Qatar.

## 5. Conclusions

Several key changes were shown to involve camels, humans, the economy, and the environment in Qatar during the last 30 years. Our study indicates that the rapid increase in camel ownership, leading to the presence of camels from different origins in a high-density environment mixed together with human and other animal species may have offered the right circumstances for the virus to spread from camels to humans. The other key changes that were described collectively contributed to this situation. Further understanding of the drivers that led to the emergence of MERS-CoV can serve as input for MERS-CoV surveillance and control measures to prevent further spread of MERS-CoV and reduce transmission from camels to humans.

## Supplementary Materials

The following are available online at http://www.mdpi.com/1999-4915/11/1/22/s1, Supplementary 1: Categories, subcategories, and data sources used for information gathering in this review; Supplementary 2: Questionnaires used for qualitative information gathering in this review.

## References

[1] Jones K.E., Patel N.G., Levy M.A., Storeygard A., Balk D., Gittleman J.L., Daszak P. (2008). Global trends in emerging infectious diseases. Nature.

[2] Coltart C.E.M., Lindsey B., Ghinai I., Johnson A.M., Heymann D.L. (2017). The Ebola outbreak, 2013–2016: Old lessions for new epidemics. Philos. Trans. R. Soc. B.

[3] Zumla A., Hui D.S., Perlman S. (2015). Middle East respiratory syndrome. Lancet.

[4] World Health Organization Middle East Respiratory Syndrome Coronavirus. http://www.who.int/emergencies/mers-cov/en/.

[5] Reusken C.B., Raj V.S., Koopmans M.P., Haagmans B.L. (2016). Cross host transmission in the emergency of MERS coronavirus. Curr. Opin. Virol..

[6] Azhar E.I., El-Kafrawy S.A., Farraj S.A., Hassan A.M., Al-Saeed M.S., Hashem A.M., Madani T.A. (2014). Evidence for camel-to-human transmission of MERS coronavirus. N. Engl. J. Med..

[7] Arwady M.A., Alraddadi B., Basler C., Azhar E.I., Abuelzein E., Sindy A.I., Sadiq B.M.B., Althaqafi A.O., Shabouni O., Banjar A. (2016). Middle East respiratory syndrome coronavirus transmission coronavirus transmission in extended family, Saudi Arabia, 2014. Emerg. Infect. Dis..

[8] Cho S.Y., Kang J., Ha Y.E., Park G.E., Lee J.Y., Ko J., Lee J.Y., Kim J.M., Kang C., Jo I.J. (2016). MERS-CoV outbreak following a single patient exposure in an emergency room in South Korea: An epidemiological outbreak study. Lancet.

[9] Alagaili A.N., Briese T., Mishra N., Kapoor V., Sameroff S.C., Wit E., Munster V.J., Hensley L.E., Zalmout I.S., Kapoor A. (2014). Middle East respiratory syndrome coronavirus infection in dromedary camels in Saudi Arabia. mBio.

[10] Lee J.Y., Kim Y., Chung E.H., Kim D., Jeong I., Lee J.Y., Kim Y., Chung E.H., Kim D., Jeong I. (2017). The clinical and virological features of the first imported case causing MERS-CoV outbreak in South Korea, 2015. BMC Infect. Dis..

[11] Müller M.A., Corman V.M., Jores J., Meyer B., Younan M., Liljander A., Bosch B., Lattwein E., Hilali M., Musa B.E. (2014). MERS coronavirus neutralizing antibodies in camels, Eastern Africa, 1983–1997. Emerg. Infect. Dis..

[12] Hassell J.M., Begon M., Ward M.J., Fèvre E.M. (2017). Urbanization and disease emergence: Dynamics at the wildlife-livestock-human interface. Trends Ecol. Evol..

[13] Jones B.A., Grace D., Kock R., Alonso S., Rushton J., Said M.Y., KcKeever D., Mutua F., Young J., McDermott J. (2013). Zoonosis emergence linked to agricultural intensification and environmental change. Proc. Natl. Acad. Sci. USA.

[14] Hui E.K. (2006). Reasons for the increase in emerging and re-emerging viral infectious diseases. Microbes Infect..

[15] Hemida M.G., Elmoslemany A., Al-Hizab F., Alnaeem A., Almathen F., Faye B., Chu D.K.W., Perera R.A.P.M., Peiris M. (2017). Dromedary camels and the transmission of Middle East respiratory syndrome coronavirus (MERS-CoV). Transbound. Emerg. Dis..

[16] Cauchemez S., Nouvellet P., Cori A., Jombart T., Garske T., Clapham H., Moore S., Mills H.L., Salje H., Collins C. (2016). Unraveling the drivers of MERS-CoV transmission. Proc. Natl. Acad. Sci. USA.

[17] Hobbs M. Divers Are a Pearl’s Best Friend: Pearl Diving in the Gulf 1840S–1930S. https://www.qdl.qa/en/divers-are-pearl%E2%80%99s-best-friend-pearl-diving-gulf-1840s%E2%80%931930s.

[18] Al Janahi B.M. National Identity Formation in Modern Qatar: New Perspective. Master’s Thesis.

[19] Crystal J. (1990). Oil and Politics in the Gulf. Rulers and Merchants in Kuwait and Qatar.

[20] World Bank Qatar Country Indicators. http://data.worldbank.org/country/qatar.

[21] Embassy of the State of Qatar in Brussels Qatar History. http://www.qatarembassy.be/QatarEmbassy/English/History.html.

[22] Dougherty R.L. (1995). Bedouins of Qatar, Klaus Ferdinand.

[23] Ministry of Development Planning and Statistics Quarterly Bulletin for Population and Social Statistics—Third Quarter 2016. https://www.mdps.gov.qa/en/statistics1/pages/lateststats/20170320.aspx.

[24] Ministry of Development Planning and Statistics Labor Force Survey 2016. http://www.mdps.gov.qa/en/statistics/StatisticalReleases/Social/LaborForce/2016/Labour_force_2016_AE.pdf.

[25] Snoj J. Population of Qatar by Nationality—2017 Report. http://priyadsouza.com/population-of-qatar-by-nationality-in-2017.

[26] Ministry of Development Planning and Statistics Population and Social Statistics 2016. http://www.mdps.gov.qa/en/statistics/StatisticalReleases/Population/Population/2016/Population_social_1_2016_AE.pdf.

[27] Qatar Tourism Authority Annual Tourism Performance Report. https://www.visitqatar.qa/corporate/planning/data-and-statistics.html.

[28] Bakri A.H. (2013). Chronic Disease Risk Factor Surveillance: Qatar Stepwise Report 2012.

[29] World Health Organization (2017). Global Health Observatory (GHO) Data: Overweight and Obesity. http://www.who.int/gho/ncd/risk_factors/overweight/en/.

[30] National Health Authority Qatar World Health Survey Qatar. https://static-content.springer.com/esm/art%3A10.1186%2F1478-7954-12-18/MediaObjects/12963_2013_244_MOESM1_ESM.pdf.

[31] World Health Organization Noncommunicable Diseases and Their Risk Factors; STEPwise Approach to Surveillance (STEPS). http://www.who.int/ncds/surveillance/steps/en/.

[32] World Bank Death Rate, Crude Death. https://data.worldbank.org/indicator/SP.DYN.CDRT.IN.

[33] Camel Racing Committee (2016). The Reports of The Camel Racing Organizing Committee.

[34] Department of Animal Resources (2016). Yearbook of Animal Statistics 2015.

[35] World Organisation for Animal Health (OIE) Animal Population; World Animal Health Information Database (WAHIS Interface)—Version 1. https://www.oie.int/wahis_2/public/wahid.php/Countryinformation/Animalpopulation.

[36] Ministry of Municipality and Environment Doha, Qatar. http://www.mme.gov.qa/cui/index.dox?siteID=2,2014.

[37] Ministry of Development Planning and Statics (2014). Environment Statistics Annual Report. 2013.

[38] Ministry of Development Planning and Statistics Environment Statistics in the State of Qatar. https://www.mdps.gov.qa/en/statistics/Statistical%20Releases/Environmental/EnvironmentalStatistics/Environment_QSA_EN_2015.pdf.

[39] Supreme Council for Environment and Natural Reserves Protected Area Action Plan 2008–2013, Conversion of Biological Diversity (CBD). https://www.cbd.int/doc/world/qa/qa-nbsap-oth-en.pdf.

[40] Elford C.J. (2013). Opportunities for the Sustainable Use of the Camel in Qatar. Master’s Thesis.

[41] Zaki A.M., Boheemen S.V., Bestebroer T.M., Osterhaus A.D., Fouchier R.A. (2012). Isolation of a novel coronavirus from a man with pneumonia in Saudi Arabia. N. Engl. J. Med..

[42] Khalfallah A.I., Lu X., Mubarak A.I.A., Dalab A.H.S., Al-Busadah K.A.S., Erdman D.D. (2015). MERS-CoV in upper respiratory tract and lungs of dromedary camels, Saudi Arabia, 2013–2014. Emerg. Infect. Dis..

[43] Badawi A., Ryo S.G. (2016). Prevalence of comorbidities in the Middle East respiratory syndrome coronavirus (MERS-CoV): A systematic review and meta-analysis. Int. J. Infect. Dis..

[44] Lu G., Wang Q., Gao G.F. (2015). Bat-to-human: Spike features determining ‘host jump’ of coronaviruses SARS-CoV, MERS-CoV, and beyond. Trends Microbiol..

[45] Letko M., Miazgowicz K., McMinn R., Seifert S.N., Sola I., Enjuanes L., Carmody A., van Doremalen N., Munster V. (2018). Adaptive Evolution of MERS-CoV to Species Variation in DPP4. Cell Rep..

[46] Adney D.R., Doremalen N.V., Brown V.R., Bushmaker T., Scott D., Wit E.D., Bowen R.A., Munster V.J. (2014). Replication and shedding of MERS-CoV in upper respiratory tract of inoculated dromedary camels. Emerg. Infect. Dis..

[47] Chu D.K.W., Hui K.P.Y., Perera R.A.P.M., Miguel E., Niemeyer D., Zhao J., Channappanavar R., Dudas G., Oladipo J.O., Traoré A. (2018). MERS coronaviruses from camels in Africa exhibit region-dependent genetic diversity. Proc. Natl. Acad. Sci. USA.

