# Supplementary material for: Drivers of MERS-CoV Emergence in Qatar
Source: Viruses. 2018 Dec 31;11(1):22. doi: 10.3390/v11010022 (PMC6356962; doi:10.3390/v11010022)
Supplement: Supplementary file 1 [file viruses-11-00022-s001.pdf]

Drivers of MERS-CoV emergence in Qatar

Annex 1: Categories, subcategories, and data sources used for information gathering in this review.

Methodology: This review aims to summarize quantitative dataset containing human, animal, and environmental factors to investigate the possible drivers that contributed to the MERS-CoV emergence in Qatar. The review mainly refers to changes in the last 30 years.

Categories	Sub-categories	Description of sub-categories	Reference
Human demography and behavior	Population	Total population	1, 2
		Gender wise population	1
		Age wise population	1, 3
		Municipality wise population distribution	32
		Population by nationality and origin	4, 5, 6
		Economically active peoples	4, 32
	Comorbidities and death	Smoking	7, 35
		Obesity	8
		Cardiovascular	7, 9, 30
		Diabetes	7, 9, 10
		Asthma	11
		Chronic Lung Disease	9
		High Blood Pressure	7, 9, 36
		Kidney Failure	9
		Chronic Liver Disease	9
		Chronic Anemia	9
		Cancer	9
		Immune Deficiency	12
		Total death and death rate	9
	Sanitation	Number of buildings connected to public sewage	26
		Number of buildings connected to water	27
		Access to drinking water	37
		Population with access to improved water source	13
		Population using improved sanitation facilities	13
	Cultural practices around camels	People living in rural and urban area	18
		Employment in agriculture	18
	Knowledge level	Educational status of population	14
		Health education	30
Economic development	Oil and GDP		16, 17, 18
International travel, commerce, sports and leisure	Tourists	Total arrival and number of tourists	15, 18
		Doha airport arriving passengers	16
		Origin of visitors	15
	Camel related travel	Cross border movement and travel of workers and owners	4
		Camel import and export	19, 23, 24
		Camel type (Race, show, and others) and origin in import	19
		Show camel events (number of shows per year)	39
Agricultural and food industry change	Camel demography	Total number and density per area	19, 29, 21
	Farming demography	Total number of farms and density per farms	19
	Camel types	Race, show, and others	22, 38
	Camel products	Production: milk and meat	22, 40
	Camels slaughtering	Total slaughterhouse and number of slaughtered animals	19, 22
	Feed import	Camel and other livestock	24
	Other livestock	Sheep, goat, cattle, and horse	40
	Climate and weather	Temperature, humidity and rainfall	25, 26
	Land use change	surface protected area and cultivable land	27, 28, 40

References:

1. Ministry of Development Planning and Statistics-Qatar. Population statistics. Available online: http://www.mdps.gov.qa/en/statistics1/pages/topicslisting.aspx?parent=Population&child=Population (accessed 20 June 1017).
2. The worldbank Data. Qatar. Available online: http://data.worldbank.org/country/qatar (accessed 23 August 2017)
3. Ministry of Development Planning and Statistics-Qatar. Qatar in figures 2010 and 2014. Available online: http://www.mdps.gov.qa/en/statistics1/pages/topicslisting.aspx?parent=General&child=QIF (Accessed 30 June 2017).
4. Ministry of Development Planning and Statistics-Qatar. Labor Force statistics. Available online: http://www.mdps.gov.qa/en/statistics1/pages/topicslisting.aspx?parent=Social&child=LaborForce (Accessed 28 June 2017).
5. Demography, migration and labour market in Qatar, GLMM - EN - No. 8/2014. Available online: http://cadmus.eui.eu/bitstream/handle/1814/32431/GLMM_ExpNote_08-2014.pdf?sequence=1 (Accessed 28 June 2017).
6. Population by nationality (Qatari / Non-Qatari) at dates of census (1970-2010). Available online: http://gulfmigration.eu/population-by-nationality-qatari-non-qatari-census-1970-2010/ (Accessed 28 June 2017).
7. Qatar Stepwise report 2012. Available online: http://www.who.int/chp/steps/Qatar_2012_STEPwise_Report.pdf?ua=1 (Accessed 12 July 2017).
8. World Health Organization. Global Health Observatory (GHO) data. Overweight and obesity. Available online: http://www.who.int/gho/ncd/risk_factors/overweight/en/ (Accessed 12 July 2017).
9. World Health Organization. Mortality database. Available online: http://apps.who.int/healthinfo/statistics/mortality/whodpms/ (Accessed 12 July 2017).
10. Diabetes Atlas. https://www.idf.org (Accessed July 12, 2017).
11. Annual mortality rate asthma Qatar. Available online: http://global-disease-burden.healthgrove.com/l/48258/Asthma-in-Qatar (Accessed 13 July 2017).
12. Ministry of Development Planning and Statistics-Qatar. Health Report 2015. Available online: http://www.mdps.gov.qa/en/statistics/Statistical%20Releases/Social/Health/2015/Health_6_2015_AE.pdf (Accessed: 12 July 2017).
13. Worldbank Improved water source access. Available online: http://data.worldbank.org/indicator/SH.H2O.SAFE.ZS?locations=QA (Accessed 30 June 2017).
14. Ministry of Development Planning and Statistics-Qatar. Population and social statistics 2011. Available online: http://www.mdps.gov.qa/en/statistics1/pages/topicslisting.aspx?parent=Population&child=Population (Accessed 25 June 2017).
15. Annual tourism performance report 2016. Available online: https://www.visitqatar.qa/corporate/statistics/2016 (Accessed 11 July 2017).
16. Ministry of Development Planning and Statistics-Qatar. Qatar in figures. Available online: http://www.mdps.gov.qa/en/statistics/Statistical%20Releases/General/QIF/Qatar_in_Figures_MDPS_AE_2011.pdf (Accessed 11 July 2017).
17. Organization of the Petroleum Exporting Countries. Annual Statistical Bulletin. Available online: http://www.opec.org/opec_web/en/202.htm (Accessed 11 July 2017)
18. The worldbank Data. Available online: http://databank.worldbank.org/data/reports.aspx?source=2&country=QAT (Accessed 29 June 2017).
19. Department of Animal Resources, Doha-Qatar
20. FAOstat compare Production, Live Animals, Qatar, Camels. Available online: http://www.fao.org/faostat/en/#compare (Accessed 10 July, 2017).
21. Animal Population Qatar. Available online: http://www.oie.int/wahis_2/public/wahid.php/Countryinformation/Animalpopulation (Accessed 30 June 2017).
22. FAOstat compare Production, Livestock Primary, Qatar, Producting animals/Slaughtered and Production Quantity, meat camel and milk whole fresh camel. Available online: http://www.fao.org/faostat/en/#compare (Accessed 28 June 2017).

23. Ministry of Development Planning and Statistics-Qatar. Foreign Trade. Available online: http://www.mdps.gov.qa/en/statistics1/pages/topicslisting.aspx?parent=Economic&child=ForeignTrade (Accessed 28 June 2017).
24. FAOstat compare Trade, Live Animals, Qatar, Import Quantitiy and Export Quantity. Available online: http://www.fao.org/faostat/en/#compare (Accessed 11 July 2017).
25. Climate Change Knowledge Portal. Available online: http://sdwebx.worldbank.org/climateportal/index.cfm?page=country_historical_climate&ThisRegion=Middle%20East&ThisCCode=QAT (Accessed 10 July 2017).
26. Ministry of Development Planning and Statistics-Qatar. Water Statistics in the state of Qatar. Available online: http://www.mdps.gov.qa/en/statistics1/pages/topicslisting.aspx?parent=Environmental&child=Water (Accessed 10 July 2017).
27. Ministry of Development Planning and Statistics-Qatar. Environmental statistics 2013 and 2015. Available online: http://www.mdps.gov.qa/en/statistics1/pages/topicslisting.aspx?parent=Environmental&child=EnvironmentalStatistics (Accessed 29 June 2017).
28. The worldbank Data. Agricultural area, rural area, urban area. Available online: http://databank.worldbank.org/data/reports.aspx?source=2&country=QAT (Accessed 10 July 2017).
29. CIA Factbook Qatar. Available online: http://www.ciaworldfactbook.us/asia/qatar.html (Accessed 19 July 2017).
30. National Health Authority (Qatar), Qatar Statistics Authority and World Health Organization. World Health Survey-2006 Qatar. Available online: http://www.biomedcentral.com/content/supplementary/1478-7954-12-18-S1.pdf (Accessed 29 July 2017).
31. Bener A, Zirie M, Musallam M, Khader YS, Al-Hamaq AO. Prevalence of metabolic syndrome according to Adult Treatment Panel III and International Diabetes Federation criteria: a population-based study. *Metabolic Syndrome and Related Disorders* 2009, 7, pp. 221—229. doi: 10.1089/met.2008.0077
32. Ministry of Development Planning and Statistics-Qatar. Population statistics, census data 2004, 2010, 2015. Available online: http://www.mdps.gov.qa/en/statistics1/pages/topicslisting.aspx?parent=General&child=Census (Accessed 24 July 2017).
33. Elford, C.J. Opportunities for the sustainable use of the camel in Qatar, MS Thesis, Virginia Commonwealth University, VCU Scholars Compass; 2013. Available online: https://core.ac.uk/download/pdf/51293060.pdf (Accessed 17 July 2017).
34. Population of Qatar by nationality - 2017 report. Available online: http://priyadsouza.com/population-of-qatar-by-nationality-in-2017/ (Accessed 25 July 2017).
35. Ministry of Development Planning and Statistics-Qatar. Global Adult Tobacco Survey 2013. Available online: http://www.mdps.gov.qa/en/statistics/Statistical%20Releases/Social/Health/Gats/2013/GATS-BOOK.pdf (Accessed 26 July 2017).
36. World Health Organization, Global Health Observatory data repository. Available online: http://apps.who.int/gho/data/node.main.A875?lang=en (Accessed 20 July 2017)
37. https://washdata.org/ (Accessed 26 July 2017).
38. Camel racing committee, Doha-Qatar.
39. Where Camels Race and Win Beauty Contests, the New York Times. Available online: https://www.nytimes.com/2017/02/10/world/middleeast/camel-beauty-contest-abu-dhabi.html (Accessed 31 July 2017).
40. Ministry of Development Planning and Statistics-Qatar. Agricultural statistics 2012, 2013, 2014. Available online: https://www.mdps.gov.qa/en/statistics1/pages/topicslisting.aspx?parent=Economic&child=Agriculture (Accessed 31 July 2017).

Annex 2: Questionnaires used for qualitative information gathering in this review

Methodology: Interviews with a group of 15 experts and stakeholders from Qatar aimed to summarize quantitative information and remaining data gaps in the quantitative dataset containing human, animal and environmental factors to investigate the possible drivers that contributed to the MERS-CoV emergence in Qatar. The question mainly refer to changes in the last 30 years.

List of the questions:

1. What are the changes with regard to camel ownership?
2. What are the changes over time with regards to behavior and living conditions of people around camels? Examples are: frequency/intensity of –camel contact, visiting camel's barns, proportion of people living with camels permanently.
3. What are the changes with regard to cultural habits involving camels, such as kissing camels and uses of camel products (meat, milk, and urine)?
4. What are the changes of the level of educational status of the camel' workers and owners?
5. What are the changes in the health education activities targeting camel workers and camel owners?
6. What has changed in the cross border movements of camels and people around camels?
7. What are the changes in movements within Qatar of camels and people around camels?
8. What has changed with regard to the demography of camels and camel farms?
9. What are the changes in camel farming practices?
10. What are the changes in the feeding of camels?
11. What are the changes in camel slaughtering practices?
12. What are the environmental changes that took place in Qatar, for example with regard to protected areas (nature conservation) and their effects on camel farming?
